# The frequency of CNVs in a cohort population of consecutive fetuses with congenital anomalies after the termination of pregnancy

**DOI:** 10.1002/mgg3.658

**Published:** 2019-04-19

**Authors:** Gorazd Rudolf, Luca Lovrečić, Nataša Tul, Nataša Teran, Borut Peterlin

**Affiliations:** ^1^ Clinical Institute of Medical Genetics (CIMG) University Medical Centre Ljubljana Ljubljana Slovenia; ^2^ Department of Perinatology Division of Gynaecology and Obstetrics University Medical Centre Ljubljana Ljubljana Slovenia

**Keywords:** congenital anomalies, copy number variations, epidemiology, molecular karyotyping, termination of pregnancy

## Abstract

**Background:**

The implementation of molecular karyotyping has resulted in an improved diagnostic yield in the genetic diagnostics of congenital anomalies, detected prenatally or after the termination of pregnancy. However, the systematic epidemiologic ascertainment of copy number variations in the etiology of congenital anomalies has not yet been sufficiently explored.

**Methods:**

Consecutive fetuses, altogether 204, with major single or multiple congenital anomalies were ascertained by using the SLOCAT registry for the period from 2011 to 2015. After excluding aneuploidies by using conventional karyotyping or Quantitative Fluorescence‐Polymerase Chain Reaction, array comparative genomic hybridization was performed for the detection of copy number variations.

**Results:**

We identified pathogenic or likely pathogenic copy number variations in 14 fetuses (6.8%); 2.9% in fetuses with isolated, and 3.9% in fetuses with multiple congenital anomalies. Additionally, aneuploidies and major structural chromosomal abnormalities were detected in 40.2%.

**Conclusion:**

Our systematic approach of ascertaining congenital anomalies resulted in explaining the etiology of congenital anomalies in 47% of fetuses after the termination of pregnancy. By using array comparative genomic hybridization, we found that copy number variations represent an important part in the etiology of multiple, as well as isolated congenital anomalies, which indicates the importance of analyzing copy number variations in the diagnostic approach of fetuses with congenital anomalies after the termination of pregnancy.

## 1 INTRODUCTION

Congenital anomalies (CAs) occur in about 2%–3% of liveborn and 20% of stillborn infants (Valduga et al., 2010). Thus, CAs are more prevalent that many chronic childhood diseases such as autism, pediatric cancers, and type 1 diabetes and are an important cause of neonatal mortality, childhood morbidity, and long‐term disability. They represent an important public health and epidemiological problem (Babkina & Graham, 2014; Di Gregorio et al., 2015; Valduga et al., 2010).

The etiology of CAs is complex; CAs can result from genetic factors, environmental factors, or a combination of both (D'Amours et al., 2012; Szczałuba et al., 2016). It is estimated that genetic factors represent an important cause of CAs and may be due to different genetic mechanisms: aneuploidies, deletions, and duplications of DNA segments (collectively known as copy number variations—CNVs), and single gene disorders (D'Amours et al., 2012; Szczałuba et al., 2016). Due to the genetic complexity, the targeted genetic diagnostics is usually problematic and challenging.

It has long been estimated that an important proportion of patients with CAs have submicroscopic chromosomal changes not detectable by conventional karyotyping (D'Amours et al., 2012; Szczałuba et al., 2016). In the last decade the availability of a comparative genomic hybridization using microarray technology (aCGH), enabled detection of CNVs down to few kb in size. The method has already replaced conventional karyotyping as a first‐tier test in the postnatal setting in patients with developmental delay, intellectual disability, autism spectrum disorders, and/or multiple congenital anomalies (Miller et al., 2010; South, Lee, Lamb, Higgins, & Kearney, 2013). Large prospective and retrospective studies showed a 5%–10% increase in detection of clinically relevant CNVs in fetuses with prenatally detected ultrasound anomalies when compared to conventional karyotyping (Lovrecic et al., 2016; Shaffer et al., 2012; Srebniak et al., 2016; Wapner et al., 2012).

Finally, retrospective cohort studies investigating fetuses with CAs after the termination of pregnancy reported a 10%–24% diagnostic yield of aCGH (Di Gregorio et al., 2015; Le Caignec et al., 2005; Valduga et al., 2010; Vialard et al., 2009). Nevertheless, these studies were mainly done on selected and small samples, resulting from experiences of individual centers.

While the contribution of chromosomal abnormalities to the etiology of CAs in fetuses after the termination of pregnancy was already systematically addressed in the EUROCAT study (Dolk, Loane, & Garne, 2010), there is no systematic epidemiologic ascertainment of CNVs in the etiology of CAs.

Therefore, in this work, we systematically evaluated the detection rate of CNVs in a cohort of consecutive fetuses with single or multiple CAs after the termination of pregnancy ascertained in the SLOCAT registry for the period from 2011 to 2015.

## 2 MATERIALS AND METHODS

### 2.1 Ethical compliance

The study was approved by The Commission of the Republic of Slovenia for Medical Ethics.

### 2.2 Patients and classification of CAs

Consecutive fetuses with major single or multiple CAs were ascertained by using the SLOCAT registry for the period from 2011 to 2015. The SLOCAT registry contains systematically collected clinical data of CAs, according to the EUROCAT methodology, classified by using the international classification of diseases and additionally, by the ontology of human phenotypes, information about pregnancies and exposures to external risk factors, as well as the results of the key genetic and non‐genetic testing.

The geographical area covered by the registry includes systematic data collection on CAs in the central Slovenian region, which covers about 40% of live births in Slovenia (6000 in year 2015). Additionally, SLOCAT covers virtually all complex cases of CAs in Slovenia, since UMC Ljubljana is a tertiary center and covers the diagnostics of complex CAs for the entire country of Slovenia.

According to the abovementioned methodology, the registry of CAs contains 204 fetuses with single or multiple CA for the aforementioned time period. CAs were detected during pregnancies which were subsequently terminated or were detected after a spontaneous abortion or intrauterine death. Each fetus was examined by a clinical geneticist and each underwent pathohistological examination. The gestational age of fetuses was from 13 to 40 weeks.

### 2.3 The genetic analysis

All 204 fetuses were included in the genetic analysis. The genetic analysis of samples was carried out sequentially in order to detect genetic abnormalities. First, conventional karyotyping or rapid test for the detection of the most common aneuploidies Quantitative Fluorescence‐Polymerase Chain Reaction (QF‐PCR) was carried out on all samples for the detection of aneuploidies and major structural chromosomal abnormalities. On samples that returned normal results we performed comparative genomic hybridization (aCGH) to assess CNVs.

### 2.4 DNA samples

DNA samples were collected by using the clinical biobank of CAs, which includes DNA samples isolated from the skin of the fetuses with CAs ascertained by using SLOCAT registry.

DNA was isolated according to manufacturer's protocol using Qiagen Mini kit (Qiagen, Valencia, CA). The quality and concentration parameters of DNA were measured with NanoDrop 2000c spectrophotometer (Thermo Fisher Scientific Inc.) and Qubit 2.0 fluorometer (Life Technologies Inc.).

### 2.5 Karyotyping and QF‐PCR

Karyotyping on GTG‐banded chromosomes (550 probes) was performed on fibroblasts. QF‐PCR for the detection of chromosomes 13, 18, 21, and sex chromosomes, was performed by using Genetic Analyser ABI 3500, Aneufast Multiplex QF‐PCR molGENTIX kit (Spain), following the manufacturer′s protocol.

### 2.6 Molecular karyotyping

Molecular karyotyping was performed by using oligonucleotide microarray with approximately 55,000 probes distributed throughout the genome (Agilent, Human CGH microarray Kit 8 × 60k, hg19 UCSC, NCBI Build 37, February 2009), which provides an average resolution of approximately 100 kb. The sample was compared with a commercial reference sample of DNA (Agilent). The results were analyzed by software CytoGenomics 3.0 (Agilent).

### 2.7 Classification of aCGH results

Called CNVs were aligned with known aberrations in publicly available databases ClinGen (http://dbsearch.clinicalgenome.org/search/), DECIPHER (Database of Chromosomal Imbalance and Phenotype in Humans using Ensembl Resources (https://decipher.sanger.ac.uk/), ClinVar (http://www.ncbi.nlm.nih.gov/clinvar/), Database of Genomic Variants—DGV (http://dgv.tcag.ca/dgv/app/home), as well as with in‐house database of detected variants and their clinical significance ascertained by trained analysts. CNVs were classified into three groups, benign, VOUS, and pathogenic, all according to ACMG Standards and Guidelines (12). Benign CNVs were those reported in abovementioned databases as benign or present in our in‐house database in more than 1% of cases. Pathogenic CNVs were either known microdeletion/microduplication syndromes or large genome copy number gain and losses, described as pathogenic in the scientific literature (PubMed). Variants classified as VOUS were either already present in cited databases as VOUS or bigger than 200 kb with OMIM gene content.

## 3 RESULTS

For the observed period from 2011 to 2015, a sample of 204 consecutive fetuses with isolated or multiple major CAs were recorded in the SLOCAT registry, according to the EUROCAT methodology.

As much as 42.7% (87) cases were with isolated CA and 57.3% (117) with multiple CAs.

**Figure 1:**
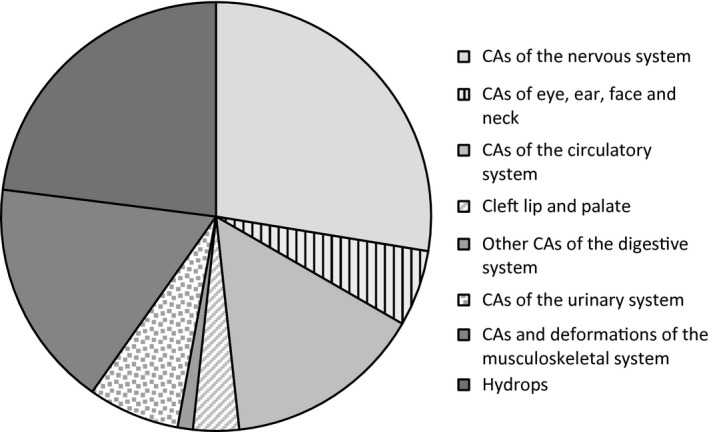
Representation of single CAs, according to the ICD10 nomenclature, in the study sample. CAs—congenital anomalies

Isolated CAs were further analyzed, depending on the type of abnormality (Figure 1).

Aneuploidies and major structural chromosomal abnormalities using conventional karyotyping or QF‐PCR were detected in 40.2% of fetuses; in isolated CAs in 37.9% and in multiple CAs in 41.9%. The most common was trisomy 18 (26.9%), followed by monosomy × (24.3%), trisomy 21 (23.1%), triploidy (16.7%), and trisomy 13 (6.4%). There was a case of mosaic trisomy 22 and a case of derivative chromosome 9 (46,XY,der(9)t(8;9)(p23;p22).ish der(9)(wcp8 + ,D8S504 + ,D9Z1 + )).

In the group of isolated CAs major chromosomal anomalies were most frequent in the cases with hydrops (85%), followed by the CAs of eye, ear, face, and neck (80%), CAs and deformations of the musculoskeletal system (40%), CAs of the urinary system (16.7%), CAs of the circulatory system (15.4%), and CAs of the nervous system (12.5%).

In fetuses with normal karyotype pathogenic or likely pathogenic CNVs were detected in 14 cases representing 6.8% of all; 2.9% represented fetuses with isolated, and 3.9% represented fetuses with multiple CAs.

Considering the group of fetuses with isolated CAs alone, pathogenic or likely pathogenic CNVs were detected in 6.9% (Table 1).

**Table 1:** Results of aCGH in a group of isolated CAs

Fetus	CA	aCGH result	Categorization
1	Agenesis of corpus callosum	arr[hg19] 2q33.3q35(208,814,372–219,814,526) × 3	Likely pathogenic
2	Occipital meningocele	arr[hg19] 18p11.32p11.21(148,963‐14,081,887) × 4	Pathogenic: 18p tetrasomy (MIM#614290)
3	Cystic hygroma	arr[hg19] 2p16.3(51,109,690–51,251,557) × 1	Likely pathogenic
4	Pulmonary valve atresia	arr[hg19] 10q23.1q23.2(82,117,139–88,710,206) × 3	Likely pathogenic
5	Short femur	arr[hg19] 6q15(91,132,714–91,651,975) × 3	VOUS
6	Hemivertebrae	arr[hg19] 16p11.2(29,673,954–30,190,568) × 1	Pathogenic; a known 16p11.2 deletion syndrome, with reduced penetrance (MIM#611913)
7	Bilateral radial aplasia	arr[hg19] 1q21.1(145,415,190–145,799,602) × 1	Pathogenic; Thrombocytopenia‐absent radius (TAR) syndrome (MIM#274000)

Fetus 1 carried an 11 Mb large duplication of chromosome 2q33.3q35. Interstitial duplications of the long arm of chromosome 2 were previously associated with intellectual disability, developmental delay, hypotonia, dysmorphic features, and different CAs; additionally, partial duplications of the long arm of chromosome 2 were described in cases with CAs of the central nervous system (Sebold et al., 2005; Usui et al., 2013).

A 142 kb large deletion spanning exons 3–5 of the NRXN1 gene was found in fetus 3. Intragenic deletions in this gene are described as a risk factor for autism and schizophrenia and are also found in individuals with developmental delay, intellectual disability, and dysmorphic features. Two prenatal cases with cystic hygroma were previously reported (Béna et al., 2013; Dabell et al., 2013; Viñas‐Jornet et al., 2014).

In fetus 4, a 6 Mb large duplication of chromosome 10q23.1q23.2 was found. Similar duplications in this region have been described in individuals with dysmorphic feature, delay in speech and development with incomplete penetrance; furthermore, CAs of the heart and the heart valves were also described (Wong et al., 2015).

In the group of fetuses with multiple CAs alone, pathogenic or likely pathogenic CNVs were detected in 6.8% (Table 2).

**Table 2:** Results of aCGH in a group of multiple CAs

Fetus	CA	aCGH result	Categorization
8	Spina bifida, ventriculomegaly, cerebellum hypoplasia	arr[hg19] 3p26.3(93,949–294,559) × 1, 3q26.1q29(166,876,046–197,837,049) × 3	Likely pathogenic
9	Omphalocele, unilateral cleft lip and palate	arr[hg19] 1q21.1q21.2(146,507,518–147,379,946) × 1,4q35.2(189,247,673–190,552,305) × 1	Pathogenic; a known 1q21 deletion syndrome, with reduced penetrance (MIM#612474)
10	Reduction anomalies of upper limbs, cleft palate	arr[hg19] 5p13.2(36,952,801‐37,024,752) × 1	Pathogenic: Cornelia de Lange syndrome (MIM#122470)
11	Micrognathia, nuchal edema, agenesis of thymus, ambivalent genitalia, anal atresia	arr[hg19] 22q11.1q11.21(17,397,498–18,628,078) × 3‐4	Pathogenic; a known 22q11.2 duplication syndrome (MIM#608363)
12	Cystic hygroma, hydrops	arr[hg19] 15q13.2q13.3(30,653,877–32,861,626) × 3	VOUS
13	Cystic hygroma, hypoplastic left heart	arr[hg19] 20p13(60,747‐748,964) × 1,20q13.13q13.33(47,912,240–62,880,583) × 3	Likely pathogenic
14	Cystic hygroma, hydrops, omphalocele, alveolar capillary dysplasia	arr[hg19] 16q24.1(86,211,031–86,649,743) × 1	Pathogenic; congenital alveolar capillary dysplasia (MIM#265380)
15	Ascites, bilateral hydrothorax, malformation of great arteries	arr[hg19] Xq13.3(74,463,757–74,651,249) × 3	VOUS
16	Micrognathia, ASD, VSD, short femurs, equinovarus	arr[hg19] 6p25.3p25.1(206,749–5,507,458) × 3 P	Likely pathogenic
17	Dilated cardiomyopathy, hydrops	arr[hg19] 16p13.11(14,910,205–16,525,348) × 3	Pathogenic; a known 16p13.11 duplicaton syndrome with reduced penetrance (ORPHA:261243)

In fetus 8 a duplication of chromosome 3q26.1q29 was found. Terminal duplications of chromosome 3q26.1q29 were associated with developmental delay, intellectual disability, CAs of the heart, cleft palate, dysmorphic features, and also CAs of CNS (Ounap, Ilus, & Bartsch, 2005; Rodríguez et al., 2014).

Additionally, a deletion of chromosome 3p26.3 was present, which, according to the literature and the size, is most likely a VOUS.

Two deletions were found in fetus 9; a deletion of chromosome 1q21.1q21.2 and a deletion of chromosome 4q35.2. First deletion represents a known 1q21 deletion syndrome with developmental delay, dysmorphic features, microcephaly, hernias, heart, skeletal, and genitourinary anomalies. According to the literature the 4q35.2 deletion is most likely a VOUS.

In fetus 13 a deletion of chromosome 20p13 and a duplication of chromosome 20q13.13q13.33 were found. Terminal deletions of the short arm of chromosome 20 were associated with developmental delay, intellectual disability, and dysmorphic features (Jezela‐Stanek, Kucharczyk, Pelc, Gutkowska, & Krajewska‐Walasek, 2013). Large terminal duplications of the long arm of chromosome 20 were previously reported in individuals with developmental delay, dysmorphic features, cleft lip and palate, CAs of the heart and thorax (Starr, Truemper, Pickering, Sanger, & Olney, 2014).

Fetus 16 carried a microduplication of chromosome 6p25.3p25. Terminal duplication of a short arm of chromosome 6 were already reported in cases with intrauterine growth retardation, choanal atresia, anomalies of the heart and heart valves, microcephaly, dysmorphic features, anomalies of the hands and feet, developmental delay, and intellectual disability (Nakane et al., 2013).

Among all 14 detected CNVs 7 were classified as pathogenic and 7 as likely pathogenic. In additional 3 cases (1.5%) the CNVs were classified as VOUS. The duplication in the region 6q15 includes only gene *MAP3K7* (OMIM*602614), linked to two autosomal, dominantly inherited human disorders, where missense mutations and in‐frame deletions were reported as causative. The reports of similar duplication are lacking in all available databases, therefore it represents a VOUS. Most likely, it is not related to a short femur, since it was inherited from an apparently healthy mother.

A 15q13.2q13.3 duplication involves eight OMIM genes, which have not been reported as causative for human disease when represented in three copies, except for *CHRNA7* (OMIM*118511). Deletion of this region represents a known risk factor for variable phenotype, including developmental delay, intellectual disability, autism, seizures, and ADHD. On the other hand, the clinical causality of duplication is still questionable, as it is inherited from a healthy parent in more than 50% of reported cases. Its penetrance is most likely influenced by other factors (Gillentine & Schaaf, 2015).

The third VOUS is a Xq13.3 duplication in a fetus with congenital heart anomaly, where part of *ZDHHC15* gene (OMIM*300576) is duplicated. This gene is a candidate gene for X‐linked mental retardation‐91, but only one family has been reported with no congenital heart anomalies. Further cases are needed to clarify the genotype‐phenotype correlation.

A cumulative additional diagnostic yield of aCGH in our study was 11.4%, when compared to conventional karyotyping.

## 4 DISCUSSION

The purpose of our study was to evaluate the contribution of CNV in the epidemiology of CAs on a systematically collected sample of consecutive fetuses with major unselected isolated or multiple CAs after the termination of pregnancy for the period from 2011 to 2015. The frequency of pathogenic or likely pathogenic CNVs in our sample was 6.8%.

Previous studies performed on fetuses with CAs after the termination of pregnancy and normal karyotypes, which were mainly focused on small (not exceeding 50 fetuses) and selected samples of fetuses with mainly multiple CAs, gave a detection rate of aCGH between 10% and 24%, showing relatively high variability (Di Gregorio et al., 2015; Le Caignec et al., 2005; Valduga et al., 2010; Vialard et al., 2009). The lowest detection rate (10%) was in a study from Valduga (2010) that included 50 fetuses with multiple CAs and the highest (24%) was found in a study from Di Gregorio et al. (2015) after analyzing 33 fetuses with isolated and multiple CAs. The discrepancies between these studies may be due to the size of the samples, different inclusion criteria, different microarray platforms, and the inclusion of VOUS among the identified CNVs (Di Gregorio et al., 2015). The diagnostic yield (11.4%) of aCGH in our study is among the lowest of the mentioned studies, which can be explained by the highest percent of isolated CAs in the sample and the fact that the sample was represented by consecutive fetuses with CAs, which were not selected by the number or the type of CAs.

Interestingly, in contrast to our expectations, the contribution of CNVs was very similar in the group of isolated and multiple CAs, which implicates the importance of applying aCGH in the diagnostic protocol also in the cases with isolated CAs.

Unlike the studies performed on fetuses after the termination of pregnancy, large prospective and retrospective studies on fetuses with prenatally detected ultrasound anomalies showed a lower, 5%–10% increase in aCGH in detection of clinically relevant copy number variation when compared to conventional karyotyping (Lovrecic et al., 2016; Shaffer et al., 2012; Srebniak et al., 2016; Wapner et al., 2012).

There is a well‐known evidence that the diagnostic yield of classical karyotype in fetuses presenting with isolated or multiple CAs is more than 18%. In retrospective series, chromosomal abnormalities were found in 2%–18% of cases when isolated and in 18%–35% when multiple CAs were prenatally detected on ultrasound (Nicholaides, Snijders, Gosden, Berry, & Campbell, 1992; Staebler et al., 2005). In our study, in addition to CNV contribution, we also analyzed the contribution of aneuploidies and major structural chromosomal anomalies in the etiology of CAs; we found them in 40.2% of cases. This result can be compared to 48% established in the EUROCAT study (Dolk et al., 2010), as the EUROCAT methodology for the assessment and classification of CAs was the same in both studies.

The strength of our study is a systematic approach on a non‐selected sample of consecutive fetuses which were thoroughly ascertained by clinical genetic and pathohistological examination through a registry of CAs rather than performing the analysis on selected cases of CAs with a high probability of chromosomal abnormality. Also, the precise characterization of the phenotype, which is an important part of the diagnostic process, can be much more detailed after the termination of pregnancy, as in our approach, compared to the period during the pregnancy.

The limitations of our study are, a still relatively small sample size due to small Slovenian population, despite a 5‐year study period, and, in terms of addressing a comprehensive genetic epidemiology of CAs, not addressing possible single gene disorders.

The systematic approach for the detection of the etiology of CAs including conventional and molecular karyotyping as employed in our study resulted in explaining the etiology in 47% of cases, which represents a significant diagnostic yield. This has important consequences for the patients, as it enables the identification of the cause of CAs and, consequently, their prevention, as well as for the understanding the genetic epidemiology of CAs and designing optimal professional and cost‐effective diagnostic algorithms for the diagnostics of CAs. Altogether, coupled with the use of standardized registries of CAs, this enables a more efficient public health approach in the process of diagnostics and prevention of CAs.

In summary, in our study, which was ascertained on a cohort population of consecutive fetuses with unselected isolated and multiple CAs after the termination of pregnancy, the frequency of CNVs was 6.8%, which implicates an important role of aCGH in the genetic diagnostics of fetuses with CAs after the termination of pregnancy.

## CONFLICT OF INTEREST STATEMENT

This research received no specific grant from any funding agency in the public, commercial, or not‐for‐profit sectors. The authors stated that there are no conflicts of interest regarding the publication of this manuscript. The authors also state that they have had full control of all primary data and that they agree to allow the Journal to review their data if requested.
